# Investigation of Mixture Modelling Algorithms as a Tool for Determining the Statistical Likelihood of Serological Exposure to Filariasis Utilizing Historical Data from the Lymphatic Filariasis Surveillance Program in Vanuatu

**DOI:** 10.3390/tropicalmed4010045

**Published:** 2019-03-08

**Authors:** Hayley Joseph, Sarah Sullivan, Peter Wood, Wayne Melrose, Fasihah Taleo, Patricia Graves

**Affiliations:** 1 The Walter and Eliza Hall Institute of Medical Research, Division of Population Health and Immunity, Melbourne, VIC 3052, Australia; 2 Department of Medical Biology, The University of Melbourne, Melbourne, VIC 3052, Australia; 3 Neglected Tropical Diseases Support Center, The Task Force for Global Health, Decatur, GA 30030, USA; ssullivan@taskforce.org; 4 College of Public Health, Medical and Veterinary Sciences, James Cook University, Cairns, QLD 4878, Australia; peter.wood@ozemail.com.au (P.W.); wayne.melrose2@bigpond.com (W.M.); patricia.graves@jcu.edu.au (P.G.); 5 Great Barrier Reef Legacy, Cairns, QLD 4877, Australia; 6 Vector Borne Disease Unit, Ministry of Health, Port Vila, Vanuatu; taleof@who.int

**Keywords:** mixture modelling, filariasis, CELISA, R statistics, elimination, surveillance, serology, Bm14

## Abstract

As the prevalence of lymphatic filariasis declines, it becomes crucial to adequately eliminate residual areas of endemicity and implement surveillance. To this end, serological assays have been developed, including the Bm14 Filariasis CELISA which recommends a specific optical density cut-off level. We used mixture modelling to assess positive cut-offs of Bm14 serology in children in Vanuatu using historical OD (Optical Density) ELISA values collected from a transmission assessment survey (2005) and a targeted child survey (2008). Mixture modelling is a statistical technique using probability distributions to identify subpopulations of positive and negative results (absolute cut-off value) and an 80% indeterminate range around the absolute cut-off (80% cut-off). Depending on programmatic choices, utilizing the lower 80% cut-off ensures the inclusion of all likely positives, however with the trade-off of lower specificity. For 2005, country-wide antibody prevalence estimates varied from 6.4% (previous cut-off) through 9.0% (absolute cut-off) to 17.3% (lower 80% cut-off). This corroborated historical evidence of hotspots in Pentecost Island in Penama province. For 2008, there were no differences in the prevalence rates using any of the thresholds. In conclusion, mixture modelling is a powerful tool that allows closer monitoring of residual transmission spots and these findings supported additional monitoring which was conducted in Penama in later years. Utilizing a statistical data-based cut-off, as opposed to a universal cut-off, may help guide program decisions that are better suited to the national program.

## 1. Introduction

Lymphatic filariasis (LF) is one of the world’s leading causes of disability [1]. This debilitating vector-borne disease, caused by the parasitic nematodes *Wuchereria bancrofti*, *Brugia malayi* and *B. timori*, afflicts more than 70 million people globally with a further 856 million people at risk [1]. In 1997, during the World Health Assembly (WHA), a resolution was approved calling for the global elimination of LF as a public health problem (WHA50.29). The resolution acknowledged the morbidity and socioeconomic costs of LF, including the general lack of awareness of the disease and the potential for its eradication. In 2000, under the direction of the WHO, the Global Program to Eliminate LF (GPELF) was developed and was based on a comprehensive strategy to rid countries of LF by 2020 [2]. The Pacific Program to Eliminate LF (PacELF) commenced in 1999 to assist the countries and territories of the Pacific area, in recognition of the unique epidemiology and historically high number of cases within this region [3].

As global prevalence declines, there has been an urgent call to validate serological diagnostic tools as a means for identifying residual endemicity, monitoring LF transmission or declaring previously endemic regions as LF-free [4]. Advances in recombinant antigen technology have allowed the exploration of using commercial kits to assess exposure in young children. Children should be anti-filarial antibody negative if transmission has been interrupted successfully [5,6,7,8,9]. One such commercially available kit is the Bm14 assay, which is an IgG_4_-specific ELISA that detects antibodies against the recombinant antigen *Brugia malayi* 14 (Cellabs Pty Ltd, Manly, Sydney, Australia), a strong immunogen [10] recognized as a potential target in LF diagnostics [11].

The Bm14 Filariasis CELISA has been utilized in multiple surveys within the South Pacific region, including American Samoa, and in Gambia [4] to assess its potential to guide programmatic decision making and future LF surveillance [9,12,13,14,15,16]. However, there has been hesitation to include these assays because of the lack of consistent, reproducible and reliable international assay standards and cut-offs to determine positivity [17]. In a multi-center evaluation of available diagnostic tools in the LF program, it was concluded that, at the time, determining a reliable cut-off for Bm14 positivity was problematic possibly due to high background Optical Density (OD) values observed when using filter paper eluates [17]. Further studies with filter paper eluates confirmed this [15] and in response, Cellabs improved their Filariasis CELISA and filter paper methodology to now be concordant with both the Center for Disease Control (CDC) in-house Bm14 assay and paired plasma [18]. This vast improvement in assay performance and reproducibility, coupled with recent sophisticated mixture modelling techniques to determine reliable cut-offs, means that the Bm14 Filariasis CELISA can again be considered for its use in the LF program. Although previous publications have alluded to the potential use of serology in the filariasis program [19], it has not yet been fully implemented as it is pending further validation.

Endemicity of LF in the South Pacific has had a long history, documented as early as 1785 in Tonga by Captain Cook [20], and the predominant vectors are the aedine (mainly *Aedes polynesiensis*) and anopheline species, the latter being prevalent in Vanuatu. Vanuatu was formerly highly endemic for LF (reviewed in [21]), however with the implementation of annual mass drug administration (MDA) from 2000 to 2004 inclusive, the antigen prevalence rate fell to 0.2% by 2006 [22]. Previous research investigating the Bm14 antibody prevalence of children in Vanuatu from a 2007 survey, which was based on previously recommended Cellabs standard OD cut-off values, indicated the strong likelihood that transmission had been interrupted [13]. These findings were supported by no detectable circulating filarial antigen (CFA) positive children aged 6 to 7 years of age [22]. An official acknowledgement of the accomplishment of LF elimination as a public health problem in Vanuatu was given by the WHO Director General and the WHO WPRO Regional Director during the 67th session of the Regional Committee Meeting held in Manila in October 2016.

Vanuatu has conducted a series of population based, sentinel site and spot-check surveys on the path to validation of elimination. A representative 90 cluster survey of all ages in 2005–2006 was conducted nationwide to determine whether MDA could be stopped and within this survey, filter paper spots were collected from children aged up to 10 years old to assess antibody status. Serological samples for antibody testing were also collected in 2008 in a targeted child survey encompassing only Ambae and Pentecost as the latter island had noticeably high antibody prevalence in children from the 2005 survey compared to the other sites. These data derived from a program in the endgame of elimination provide ideal samples to investigate the validity of the antibody cut-offs that were used in this present study.

The aim of the current study was to apply new statistical tools (mixture modelling) to define appropriate cut-offs for positivity in a situation where the country was later validated as having eliminated LF as a public health problem. Based on the results observed, these model algorithms could be implemented in future so that serological tools can have a role in guiding programmatic decision making and supporting surveillance.

## 2. Materials and Methods

### 2.1. Survey Sites for the 2005 TAS1/C Survey in Vanuatu

In 2005/2006, Vanuatu completed their C survey, referred to as the Transmission Assessment Survey (TAS) 1, inclusive of all ages, and determined that the threshold for stopping MDA had been reached based on the prevalence of circulating filarial antigen (CFA) and microfilaremia (Mf) [21]. Herein, this survey is referred to as the 2005 TAS1. Although no antigen positive children were detected in this survey, blood was also dried onto filter spots (DBFS) from children aged up to 10 years old. These DBFS were shipped to James Cook University (JCU), Australia to be eluted and tested for anti-filarial antibodies using the commercially available Filariasis CELISA kit as per manufacturer’s instructions (Cellabs Pty Ltd, Australia). These tests were undertaken in 2007 and results were reported to the Ministry of Health (MoH). At this time, antibody positivity was reported if the OD value was ≥0.400, as recommended by Cellabs. In this current study, these historical raw OD values from the 2005 TAS 1 (*n* = 1027) were utilized (Table 1). Table 1 also outlines the prevalence reported at the time, based on the original cut-off value from Cellabs referred to herein as the “original cut-off”.

### 2.2. Survey Sites for the 2008 Targeted Child Survey in Vanuatu

In 2008, a targeted survey was carried out separate to the requirements of the TAS 2 WHO LF program. This targeted survey was designed to specifically assess if there was exposure to LF utilizing Bm14 antibody serology and, therefore, ongoing transmission in children aged up to 10 years old using the Filariasis CELISA (*n* = 187). This survey predominantly encompassed Pentecost as a known hot-spot from previous surveys (see Table 1). In this smaller survey, DBFS were collected and shipped to JCU for testing, which was completed in 2008, and results were reported to the LF program manager at the MoH. Antibody positivity was determined as OD values ≥0.400 (original cut-off). In this current study, these historical raw OD values from the 2008 targeted survey (*n* = 187) were utilized (Table 2). Table 2 also outlines the prevalence reported at the time (original cut-off value).

### 2.3. Human Ethics

The study was conducted under human ethics approval number H1423, as approved by the James Cook University Research Human Ethics Committee. This study was also approved by executives from the Vanuatu Ministry of Health.

### 2.4. Filariasis CELISA

Samples were eluted and anti-Bm14 IgG_4_ was detected using the Filariasis CELISA (Cellabs Pty Ltd, Manly, Sydney, Australia) according to the manufacturer’s instructions and as previously described [15]. Briefly, one protrusion of filter paper was eluted overnight at 4 °C and assayed the following day. Plates were read at a dual wavelength of 450 nm and 650 nm with a Multiskan EX Type 355 Primary V 2.1 (Pathtec, Victoria, Australia). At the time, negative samples were defined as an OD value of <0.260, positive samples were ≥0.400 and those between these values were repeated. If <0.400, they were considered negative. These cut-offs and repeats were according to the manufacturer’s instructions at the time.

For the current study, the Filariasis CELISA was not repeated on samples; the stored historical OD values were utilized. Negative OD values were reported if the background absorbance was greater than the sample absorbance. Within the current study, the results are compared to the original cut-off.

### 2.5. Mixture Modelling Statistical Methods

There is no gold standard for Bm14 antibody serology and, therefore, there is no way to precisely measure a “true” positive antibody response. For the current study, an “antibody positive” observation is one whereby the OD value falls above the specific stated threshold.

To this end, mixture modelling uses probability distributions to enable the user to identify potential subpopulations of positive and negative results and to set a cut-off that best distinguishes between these two groups. Utilizing these probability distributions, mixture modelling can also define a range around the cut-off where the probability of misclassification is above an acceptable level and, hence, the result is best considered to be “indeterminate”.

Mixture modelling was conducted to determine cut-offs and indeterminate ranges for the datasets from the 2005 and 2008 surveys. Models were parameterized to allow for one through five components (also known as “subpopulations”) to be fit using either normal or skew-normal distributions. It should be noted that while mixture modelling is a robust statistical technique, the sample sizes required are larger than are needed for more common statistical techniques. In particular, mixture modelling using the skew-normal distribution relies on asymptotics to calculate standard errors, which may not be reliable unless the sample size is large [23]. The necessary sample size for mixture modelling is highly dependent on the characteristics of the underlying subpopulations, so there is not an explicit rule or formula to determine what constitutes a prohibitively small sample size. However, in papers which examine mixture model performance, samples of less than 100 are rarely used when modelling normal distributions and samples of less than 200 are rarely used when modelling skew-normal distributions [24,25,26]. For the majority of these studies, utilizing either normal or skew-normal distributions, there are over 1000 samples [24,25,26]. Since the sample size of the 2008 survey (*n* = 187) approached the minimum sample used in skew-normal examples, we opted to still utilize the skew-normal distribution, however to apply a higher level of scrutiny to the results of skew-normal models for this dataset.

Taking this into consideration, after models were parametrized, the best fitting model was chosen by optimizing the Bayesian Information Criterion (BIC). If a two-component model was optimal, the cut-off was set deterministically at the point where the conditional probability of belonging to either component was equally likely (referred to herein as the “absolute” cut-off). If the optimal model had only one component, this would indicate that all of the observations are part of the same subpopulation (e.g., all negative) and, hence, a cut-off is not needed. Conversely, if the optimal model had more than three components, the user must choose between which two components to set the cut-off, based on biological knowledge, historical context or a prior knowledge of plausible cut-off values. Using either the deterministic or user-informed cut-off, an indeterminate range was then calculated such that observations which could not be classified to the positive or negative component with greater than 80% certainty were labeled as “indeterminate”. Put another way, if a point falls right on the absolute cut-off, there is a 50% chance of misclassification (as the probability of belonging to either component is equally likely by definition); if a point falls right on the 80% bound of the indeterminate range, it has a likelihood of misclassification of 20%. For example, if an absolute cut-off value is 0.317 and the indeterminate range is 0.245–0.38, an OD value falling between 0.317 and 0.38 is 50–80% certain to be positive and an OD value falling between 0.245 and 0.317 is 50-80% certain to be negative.

For this current study, where the goal was to maximize sensitivity to improve the chances of finding positive cases for surveillance, the lower threshold of the 80% range was chosen as the cut-off (the “lower 80% threshold”). Therefore, when using the 80% lower threshold, the positive category includes those samples within the indeterminate range, giving a less stringent cut-off OD value for positivity. This will increase the sensitivity of the test by decreasing the threshold based on the mixture model, suggesting the potential to pick up more signs of exposure. However, increasing sensitivity will be at the expense of specificity (i.e. inclusion of a higher proportion of “false” positives) as any value between the lower 80% threshold and the absolute cut-off is only 20–50% likely to be a true positive.

All analyses were undertaken using R version 3.5.2. The script utilized to generate the cut-off and indeterminate ranges can be found at: https://github.com/sms164/cut-off/blob/master/Cut-off_Algorithm.R. A step-by-step guide can be found in Figure S1.

### 2.6. Prevalence Mapping

Maps were constructed using ESRI ArcGIS mapping software using country boundary shapefile data from Thematic Mapping [27] available under the Creative Commons License and Esri Oceans Basemap [28].

## 3. Results

### 3.1. Spread of the OD Values across Both Years; 2005 and 2008.

**Figure 1:**
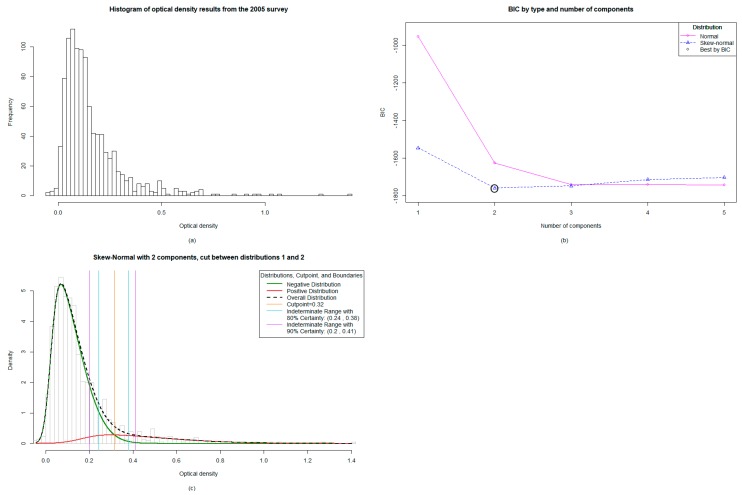
Mixture modelling analysis for the 2005 Transmission Assessment Survey/C Survey in Vanuatu: (**a**) The distribution of data from the 1027 samples was significantly right skewed, (**b**) The algorithm identified the two-component skew-normal model as optimal, (**c**) When analyzed, the two-component model showed an absolute cut-off value of 0.316 and an indeterminate range with 80% certainty falling between 0.244 and 0.38.

**Figure 2:**
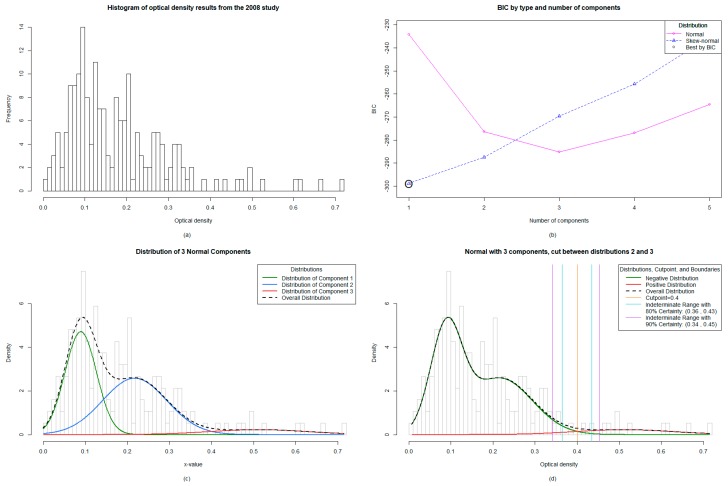
Mixture modelling analysis for the 2008 Targeted Child Survey in Vanuatu: (**a**) The distribution of data from the 187 samples was significantly right skewed, (**b**) The algorithm identified the one-component skew-normal model as optimal, indicating only one population in the sample set (negatives), (**c**) Implementation of the normal distribution identified a three-component model by BIC (Bayesian Information Criterion) best fit, (**d**) The cut-off values were set between the second and third distribution of data, giving an absolute cut-off value of ≥0.401 and a lower 80% threshold value of ≥0.365.

**Figure 3:**
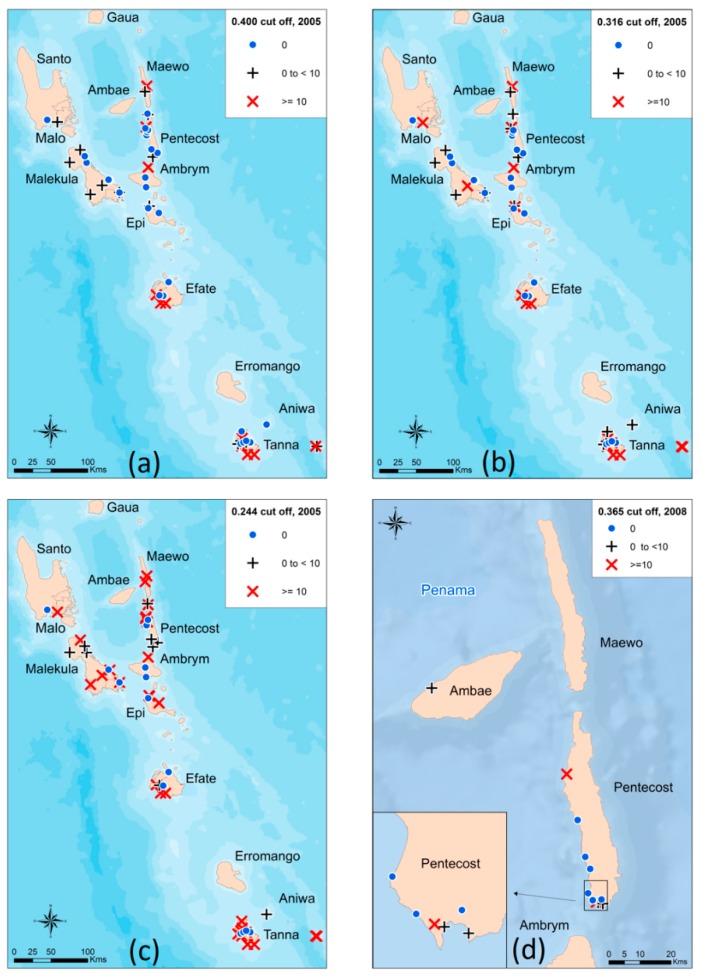
Mapping of antibody prevalence estimates for 2005 and 2008 based on the Bm14 Filariasis CELISA: (**a**) Estimates of antibody prevalence for 2005 when using the original cut-off of ≥0.400, (**b**) Estimates of antibody prevalence for 2005 when using the absolute cut-off of ≥0.316, (**c**) Estimates of antibody prevalence for 2005 when using the 80% lower threshold cut-off of ≥0.244, (**d**) Estimates of antibody prevalence for 2008 when using the 80% lower threshold cut-off of ≥0.365.

The 2005 survey included Bm14 antibody data from 1027 participants aged up to 10 years old. The ELISA results, provided as OD values, ranged from −0.044 to 1.403, and were significantly right skewed (Figure 1a). The targeted child survey completed in 2008 included Bm14 antibody data from 187 participants who were also aged up to 10 years old. The ELISA OD values ranged from 0.007 to 0.715 and, like the data from 2005, were significantly right skewed (Figure 2a). For both sets of data, the original cut-off used was ≥0.400 and the prevalence from 2005 (Table 1) was also mapped accordingly (Figure 3a). These original results were reported to the MoH.

### 3.2. The Mixture Modelling Algorithm for the 2005 TAS 1 Survey Data

The mixture modelling algorithm identified the two-component skew-normal model as optimal (Figure 1b), with the absolute cut-off falling at ≥0.316 and an indeterminate range with 80% certainty falling between 0.244 and 0.38 (Figure 1c). This indicates that OD values falling within this indeterminate range are 80% likely to be positive, with a 20% chance of misclassification statistically. The two-component model means that there are predominantly two populations: positive and negative.

### 3.3. Mixture Modelling Algorithms suggest a Higher Antibody Prevalence of Children from the TAS1 Survey in 2005 Utilizing both the Absolute Cut-off Value and the Lower 80% Certainty Cut-off Value

When using the absolute cut-off value of ≥0.316, the overall antibody prevalence was 9.0% (positives *n* = 92) compared to 6.4% from the original ≥0.400 (positives *n* = 66; Table 1). This data supported previous findings that there were problem areas in Pentecost (Figure 3a and 3b). When re-classifying those observations falling within the indeterminate range (*n* = 86) into positives by utilizing the lower 80% threshold to maximize sensitivity at the cost of specificity, the antibody prevalence increased to 17.3%, with 178 children classified as antibody positive. The antibody prevalence per village was subsequently mapped (Figure 3c).

### 3.4. The Mixture Modelling Algorithm for the 2008 Targeted Child Survey Data

The mixture modelling algorithm identified the one component skew-normal model as optimal, which indicated that a single subpopulation gave rise to the observed data (i.e. all negative) (Figure 2b). While it is possible that the single component model represents the underlying truth of the data, the potential instability of skew-normal mixture modeling on a dataset as small as the 2008 study (*n* = 187) led us to exclude mixture models utilizing the skew-normal distribution and to only consider models which utilized the more robust normal distribution. Of the normal distribution models, a three-component model fit best by BIC (Figure 2c). When choosing whether to set a cut-off between the first and second or second and third distributions, we chose to set it between the second and third, giving a higher cut-off, as this was consistent with our understanding of the epidemiology since there were no antigen positive children at the time. Given the lack of a gold standard, we assumed in a near elimination setting that there are different noisy negative phenotypes. For example, those that have never been exposed or those who had early life exposures that never mounted a true infection and have since waned; a plausible phenotypic explanation for the three-component optimal model. This yielded a cut-off falling at ≥0.401 and an indeterminate range with 80% certainty falling between 0.365 and 0.43 (Figure 2d).

### 3.5. Antibody Prevalence from the 2008 Survey Remains Unchanged with New Mixture Modelling Absolute Cut-off Value and Increases Slightly with Lower 80% Certainty Threshold Cut-off Value

The antibody prevalence remained unchanged when utilizing the absolute cut-off value of ≥0.401 (5.9%) compared to the original manufacturer’s value of ≥0.400 (5.9%) (positives *n* = 11). When re-classifying the indeterminate range (*n* = 1) into positives by utilizing the lower 80% threshold to maximize sensitivity at the cost of specificity, the antibody prevalence increased to 6.4%, with 12 children classified as antibody positive. The antibody prevalence per village was subsequently mapped (Figure 3d). The included an antibody positive child who was located in the village of Londar, Pentecost.

## 4. Discussion

The lymphatic filariasis (LF) program is reaching a crucial turning point whereby it has become critical to employ diagnostic tools that are capable of reliably informing programmatic decisions concerning the cessation of mass drug administrations (MDAs) and, subsequently, the validation of the elimination of LF as a public health problem. In order to do so, diagnostic tools need to sensitively detect ongoing transmission and, most importantly, be internationally standardized and reproducible. Given the difficulty of very low prevalence in young children and large sample sizes needed to detect low antigen prevalence in the endgame, the current focus is shifting towards considering antibody serology as a tool in these end-stages. Advances in Bm14 antibody testing reliability [18] and review of antibody results in formerly endemic countries [4] and at repeat time points [29] has provided evidence for utility of antibody serology in LF programmatic decisions. However, it has also been recognized that providing only one standard cut-off value by the manufacturer may not be suitable in varying epidemiological settings, and previous ROC curves could not adequately solve this dilemma [17]. It is known that there are different species causing LF, different co-infections and different genetic profiles, all of which could influence the antibody response in a population. The current Filariasis CELISA is based on reporting OD values alone, not concentrations obtained from the positive control curve. Recent advances in mixture modelling allow for the non-biased estimation of appropriate cut-offs for positivity in serological tests with continuously distributed outputs (e.g., OD values from ELISAs). To this end, we applied this methodology to historical OD values to investigate if mixture modelling could solve the issue of needing relevant dynamic cut-off values depending on the epidemiological setting at the time of sampling rather than a consistent standard cut-off value.

Mixture modelling involves the input of the range of values, in this case OD values, to assess the distribution of data and then consequently calculate an unbiased model such as a two-component (2005 data; Figure 1b) or three-component (2008 data; Figure 2c,d) model. Once the predicted model is calculated, it allows the LF program manager to choose an appropriate cut-off based on the distribution of data and the stringency required for the classification of positives. For example, when endemicity is high, and therefore so is exposure, it may be pertinent to have a stringent high “absolute” cut-off OD value with fewer positives in order to target areas of high transmission (and, therefore, higher OD values and, thus, higher antibody titers). Previous studies have indicated a correlation between higher OD values and ongoing transmission [13], suggestive of a correlation between antibody titer and OD value, which is the general application method for ELISAs [30]. In this instance, the absolute cut-off value identified by the mixture model may be adequate, which was ≥0.316 (2005 data) and ≥0.401 (2008 data).

As prevalence declines and surveillance and monitoring become important, the cut-off value required could be less stringent to allow the LF program manager the flexibility of not excluding positive children and, therefore, adequately identifying areas before there is high resurgence. Therefore, when not wanting to miss these potentially positive children, one could set the cut-off value at the lower 80% certainty range of ≥0.244 (2005 data) or ≥0.365 (2008 data). This is at the expense of specificity and will erroneously misclassify a proportion of negative samples as positive, which is a trade-off for higher sensitivity. After applying these methods to serological antibody data from Vanuatu around the time of post MDA surveillance, it was shown that the absolute cut-off calculated for the 2005 dataset was lower than previously recommended for the Bm14 ELISA (≥0.316 compared to ≥0.400). Thus, antibody prevalence in children was likely higher than previously appreciated. Furthermore, the lower 80% certainty threshold of ≥0.244 increased the antibody prevalence to 17.3% compared to both the original recommended cut-off and the absolute cut-off. It may be pertinent to use the lower 80% threshold in the first TAS in order to increase confidence that the program manager is not excluding any positive cases after completion of MDAs. These data concurred with previous conclusions of ongoing transmission in the province of Penama (encompassing both Ambae and Pentecost). In terms of relevance for the LF program, if the lower 80% certainty threshold for antibody prevalence determined by mixture modelling was historically available, it may have provided an earlier warning system prior to TAS4 undertaken in 2012, whereby 2 CFA positive children were detected [22]. Nonetheless, these results do indicate the necessity for ongoing close monitoring in Penama to ensure against resurgence.

Although there were no major differences observed, with different thresholds for antibody prevalence estimates in 2008, there were antibody positive children detected in the province of Penama. If the lower 80% threshold was utilized, one extra antibody positive child was identified in the village of Londar. Interestingly, Londar had an overall CFA prevalence of 6.1% of all ages in 2005, however no antigen positive children in 2008 [22]. Subsequent TAS surveys in Vanuatu have passed and the country was validated for elimination.

For the current study, the consistent village between both years was Namaram, Pentecost. The relevance of utilizing the lower 80% threshold is clearly highlighted for this village, since the 2005 data revealed prevalence values of 23.5%, 29.4% and 52.9%, respectively, for the different thresholds (Cellabs original, new absolute and new lower 80% threshold). In 2008, the data calculated a consistent prevalence across all three cut-off values of 41.7%. If only the original cut-off recommended by the manufacturer was employed, the prevalence almost doubles from 2005 to 2008. This sharp increase in potential ongoing transmission may be difficult to control quickly, whereas if the new absolute or lower 80% threshold was utilized in 2005, the antibody prevalence of 52.9% at the time may have flagged a troublesome area for concentrated efforts.

Future studies to evaluate the feasibility of utilizing the Filariasis CELISA programmatically should compare the antibody prevalence determined appropriate by the mixture modelling algorithm to other diagnostic assays that assess ongoing transmission. Previous work has compared xenomonitoring (mosquito infection rates) to antibody prevalence [31], as well as CFA prevalence in children (immunochromatographic test or Og4C3 ELISA) and antibody prevalence [29,32]. Application of the mixture modelling algorithm to these historical data, which have other measures of transmission, would be useful for comparison. However, a limitation of this technique would be the requirement for knowledge of the statistics and the outputs of the mixture modelling algorithms in order to implement appropriate cut-off values for each study.

In conclusion, mixture modelling is a powerful statistical tool allowing objective and dynamic cut-off values to be utilized depending on the distribution of values at the time of survey. Using a statistical data-based cut-off, as opposed to a universal cut-off, may help lead to program decisions that are better suited to the national program and will allow international standardization for reported results for reliable reproducibility. An understanding of the trade-off between sensitivity and specificity must be clear, as choosing an appropriate cut-off value will create false positive or negative results depending on the stringency chosen. Future studies using this technique can be undertaken in different epidemiological settings to include samples that are LF positive and using all ages to investigate the reproducibility of the algorithms. In the current study, our data strongly advise for continued close monitoring of the province of Penama in Vanuatu.

## Figures and Tables

**Table 1:** 2005 TAS (Transmission Assessment Survey)1/C survey sites, number of samples taken from children aged up to 10 years of age and the prevalence of Bm14 antibody as determined based on cut-off 1 (original cut-off).

Province	Island	Village	Number of Samples	% Prevalence
Malampa	Ambrym	Maat	20	10
		Nova—Londre	27	11.1
		Sameou	26	0
		Maranata	5	0
	Malekula	Dravai/Lamap	53	1.9
		P.R.V.	36	0
		Wala Mainland	24	0
		Pikaier	19	5.3
		Lawa	14	7.1
		Melken	19	5.3
		Pandeur	16	0
	Paama	Liro	26	3.8
Sanma	Santo	Malotau	16	0
		Tanavoli	19	5.3
Penama	Ambae	Lovositarivue	2	0
	Maewo	Naviso	31	38.7
		Rembu	6	0
		Nasawa	14	7.1
	Pentecost	Baie Barrier	39	0
		Abwatunbuliva	19	5.38
		Lalbung	18	5.6
		Laone	19	0
		Leravinanposvi	21	0
		Likasak	4	0
		Melsisi	4	0
		Namaram	17	23.5
		Pannas	11	0
Shefa	Efate	Erakor	17	17.6
		Eratap	14	14.3
		Mele	15	0
		Paonangisu	15	0
		Rango Rango	22	0
	Epi	Brisbane	11	0
		Lamenu Bay	26	3.8
		Mate	9	0
Tafea	Aniwa	Ikaokao	39	0
	Futuna	Iasoa	9	11.1
		Matangi	15	6.7
	Tanna	Eniai	20	0
		Fetukai	30	20
		Ipai	14	7.1
		Ikakahak	30	43.3
		Ikapow	1	0
		Imafen	48	0
		Imereupow	50	4
		Isiai	8	12.5
		Lahwenuwi	10	0
		Lenaken	24	4.2
		Lenawawa	16	0
		Lounapaio	9	11.1
		Lounapkao	10	0
		Lowkwaria	40	7.5
		**TOTAL**	**1027**	**6.4**

**Table 2:** 2008 Targeted Child Survey, number of samples taken from children aged up to 10 years of age and the prevalence of Bm14 antibody as determined based on cut-off 1 (original cut-off).

Province	Island	Village	Number of samples	% prevalence
Penama	Ambae	Nanako	14	7.1
	Pentecost	Bai Martelli	17	5.9
		Hot Wota	1	0
		Londar	29	10.3
		Lonlebule	2	0
		Namaram	12	41.7
		Pannas	1	0
		Point Cross	74	1.4
		Ranliae	4	0
		Ranputor	1	0
		Vansemakul	1	0
		Wanur	31	0
		**TOTAL**	**187**	**5.9%**

## Supplementary Materials

The following are available online at https://www.mdpi.com/2414-6366/4/1/45/s1, Figure S1: Step-by step guide to mixture modelling algorithms.
