# Supplementary material for: Investigation of Mixture Modelling Algorithms as a Tool for Determining the Statistical Likelihood of Serological Exposure to Filariasis Utilizing Historical Data from the Lymphatic Filariasis Surveillance Program in Vanuatu
Source: Trop Med Infect Dis. 2019 Mar 8;4(1):45. doi: 10.3390/tropicalmed4010045 (PMC6473238; doi:10.3390/tropicalmed4010045)
Supplement: Supplementary file 1 [file tropicalmed-04-00045-s001.pdf]

Tropical Medicine and Infectious Disease

MDPI

Article

Investigation of Mixture Modelling Algorithms as A Tool for Determining Statistical Likelihood of Serological Exposure to Filariasis Utilizing Historical Data from the Lymphatic Filariasis Surveillance Program in Vanuatu

Hayley Joseph [1,2,*], Sarah Sullivan [3], Peter Wood [4,5], Wayne Melrose [4], Fasihah Taleo [6] and Patricia Graves [4]

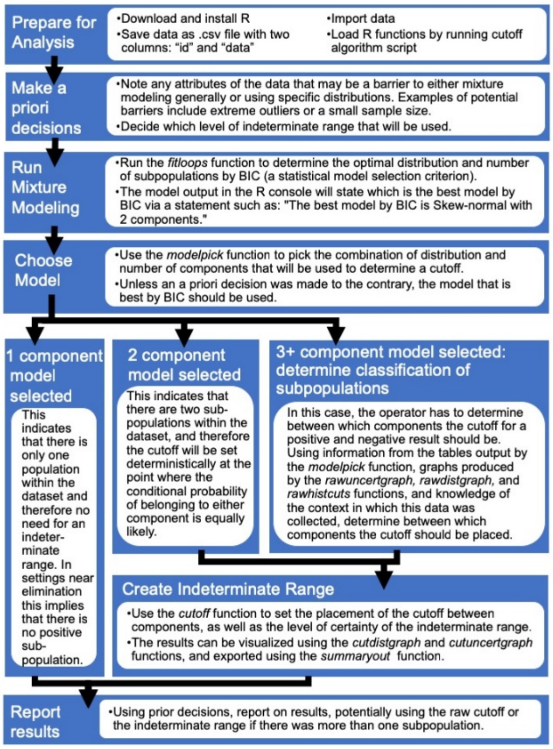

Figure S1. Step-by step guide to mixture modelling algorithms.
